# Autophagy regulates the cancer stem cell phenotype of head and neck squamous cell carcinoma through the noncanonical FOXO3/SOX2 axis

**DOI:** 10.1038/s41388-021-02115-7

**Published:** 2021-11-19

**Authors:** Yang Chen, Hui Zhao, Weilian Liang, Erhui Jiang, Xiaocheng Zhou, Zhe Shao, Ke Liu, Zhengjun Shang

**Affiliations:** 1 The State Key Laboratory Breeding Base of Basic Science of Stomatology, Hubei Province & Key Laboratory of Oral Biomedicine (Wuhan University), Ministry of Education (Hubei-MOST KLOS & KLOBM), Wuhan, China; 2 Department of Oral and Maxillofacial-Head and Neck Oncology, School & Hospital of Stomatology, Wuhan University, Wuhan, China

**Keywords:** Cancer stem cells, Autophagy

## Abstract

Autophagy is an essential catabolic process that orchestrates cellular homeostasis and plays dual roles in tumor promotion and suppression. However, the mechanism by which autophagy affects the self-renewal of cancer stem cells (CSCs) remains unclear. In this study, we investigated whether autophagy activation contributes to CSC properties of head and neck squamous cell carcinoma (HNSCC). The results showed that the autophagy level and CSC properties of HNSCC cells were elevated in response to several adverse conditions, including treatment with cisplatin, starvation, and hypoxia. Pretreatment with autophagy inhibitors, such as 3-MA and chloroquine, diminished the CSC properties acquired under adverse conditions. In addition, the isolated CSCs were endowed with stronger autophagic activity than non-CSCs, and the CSC properties were dampened when autophagy was inhibited either by 3-MA, chloroquine, or Beclin1 knockdown. Notably, the tumor-initiating activity of CSCs was decreased upon knocking down Beclin1. Further study revealed that FOXO3, a substrate for autophagy, was enriched in the nucleus of cells with lower autophagy levels. Nuclear FOXO3 directly bound to the promoter region of SOX2 and negatively regulated its transcriptional activity. Overexpression of FOXO3 decreased the expression of SOX2 and thereby impaired the CSC phenotype both in vitro and in vivo. Taken together, our findings suggest that the activation of autophagy is essential for the acquisition of CSC properties in adverse conditions and the self-renewal of CSCs. We clarify the role of autophagy in regulating the CSC phenotype and demonstrate that the noncanonical FOXO3/SOX2 axis is the intrinsic regulatory mechanism.

## Introduction

Worldwide, head and neck squamous cell carcinoma (HNSCC) is the sixth most common cancer, and over 600,000 new cases are reported annually [1]. Despite great improvements in diagnosis and therapy, the survival rate of HNSCC patients is unsatisfactory due to high recurrence and metastasis rates [2]. Cancer is a heterogeneous cell group composed of differentiated cells and tumor-initiating cells. Tumor-initiating cells are also referred to as cancer stem cells (CSCs), which possess the abilities of symmetrical self‐renewal and asymmetrical differentiation. CSCs have been proven to be highly resistant to traditional chemoradiotherapy and can survive better than differentiated cells in unfavorable survival circumstances [3]. CSCs exist in HNSCC and play vital roles in tumor occurrence and development [4–6]. Therefore, finding effective methods to eliminate CSCs is important for HNSCC therapy. Macroautophagy (generally referred to as autophagy) is a catabolic process in which eukaryotic cells degrade damaged and superfluous organelles and proteins. Autophagy plays a crucial role in maintaining cell homeostasis in adverse conditions, such as starvation, hypoxia, and chemoradiotherapy [7, 8]. The roles of autophagy in regulating cancer progression are complex and contradictory. The fate of cancers determined by autophagy depends on the type, stage, and phenotype of tumor cells. In the initial tumor stage, autophagy can eliminate misfolded proteins and damaged organelles, thus maintaining cellular homeostasis and reducing genome mutations. However, in established tumors, autophagy can fulfill the anabolic needs of tumor cells and enhance their resistance to diverse unfavorable survival circumstances [9]. Recently, autophagy has been characterized as an intrinsic feature for the maintenance of stemness in various cancers [10]. Targeting autophagy affects the self-renewal, chemoresistance, and survival of CSCs both in vitro and in vivo [11]. However, the role of autophagy in CSCs in HNSCC remains unclear.

FOXO3, also known as FOXO3a, is a member of the FOXO subfamily. FOXO3 can mediate various pathophysiological processes, including proliferation, apoptosis, cell cycle progression, longevity, antioxidative stress responses, antiirradiation responses, DNA damage, and carcinogenesis [10, 12, 13]. Most studies have demonstrated that FOXO3 acts as a tumor suppressor, and emerging evidence also indicates that FOXO3 plays a promotive effect in some cancers [14]. Moreover, FOXO3 is closely related to stem cell-like properties in various cancers [15, 16]. Recently, an interesting mechanism was uncovered: FOXO3, which can regulate the transcription of autophagy-related genes, is itself degraded by autophagy [17]. Consequently, it is necessary for us to explore the relationship between autophagy, FOXO3, and CSCs in HNSCC.

In this study, we investigated the role of autophagy in adherent HNSCC cancer cells and CSCs. We also revealed the mechanisms of autophagy in regulating the CSC phenotype. Our results provide a potential therapeutic method for targeting CSCs in HNSCC.

## Results

### The autophagy level of HNSCC cells increases under adverse conditions

**Fig. 1:**
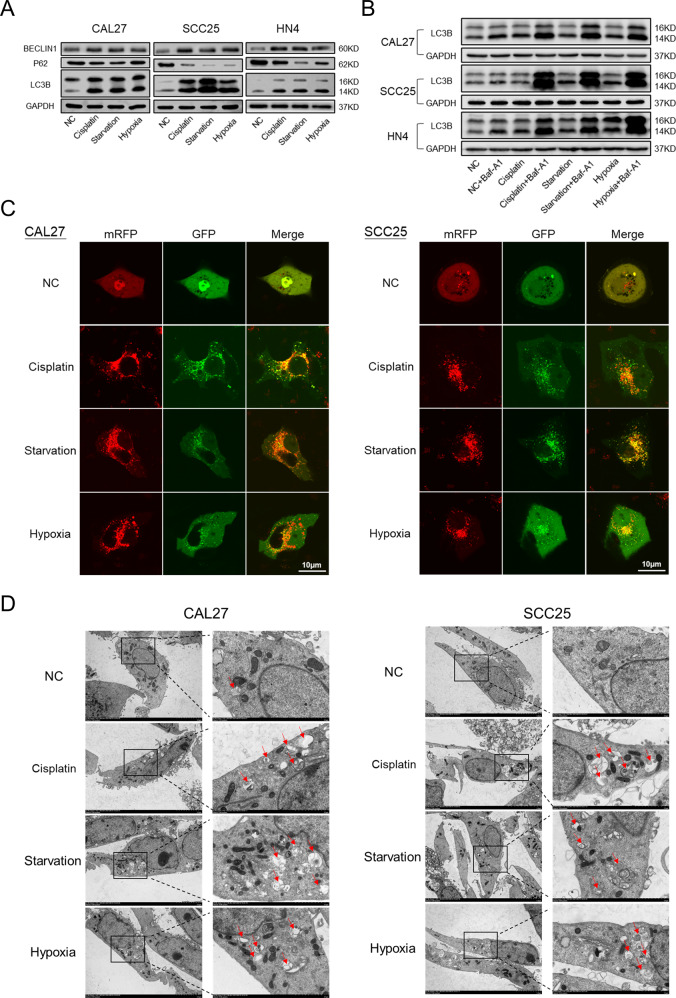
The autophagy level of HNSCC cells increases under adverse conditions. **A** Western blot was performed to determine the expression of Beclin1, p62, and LC3B in CAL27, SCC25, and HN4 cells when treated with cisplatin, starvation, and hypoxia. **B** Western blot was performed to determine the expression of LC3B in the presence and absence of Bafilomycin A1. **C** Immunofluorescence was used to observe the autophagic flux when cells were treated with cisplatin, starvation, and hypoxia. Scale bars, 10 μm. **D** Transmission electron microscope was conducted to observe autophagosomes in cells treated with cisplatin, starvation, and hypoxia.

Autophagy is essential for the homeostasis and survival of cells in adverse conditions [18]. In our research, we found that several autophagy-related proteins, such as Beclin1, p62, and LC3B, changed significantly in CAL27, SCC25, and HN4 cells treated with cisplatin (30 μM, 12 h), starvation (Hank’s balanced salt solution, 8 h), and hypoxia (1% O_2_, 72 h) (Fig. 1A). LC3B rapidly degraded during autophagy and did not indicate total autophagic flux at certain time points. Bafilomycin A1, an autophagy inhibitor at late stages, was used to block autophagic flux. The amount of LC3B was determined in the presence and absence of bafilomycin A1 (Fig. 1B). We verified that tumor cells possessed elevated autophagic flux in adverse conditions. Then, mRFP-EGFP-LC3 adenovirus was used to transfect CAL27 and SCC25 cells. The results showed that cisplatin, starvation, and hypoxia induced autophagic flux in CAL27 and SCC25 cells (Fig. 1C). In addition, transmission electron microscopy (TEM) was used to identify autophagosomes in CAL27, SCC25, and HN4 cells. Electron micrographs showed that the formation of autophagic vesicles increased in tumor cells after treatment with cisplatin, starvation, and hypoxia (Fig. 1D). Collectively, these data indicated that HNSCC cells had elevated autophagy levels in adverse conditions.

### Autophagy activation is essential for the acquisition of CSC properties in cells cultured in adverse conditions

**Fig. 2:**
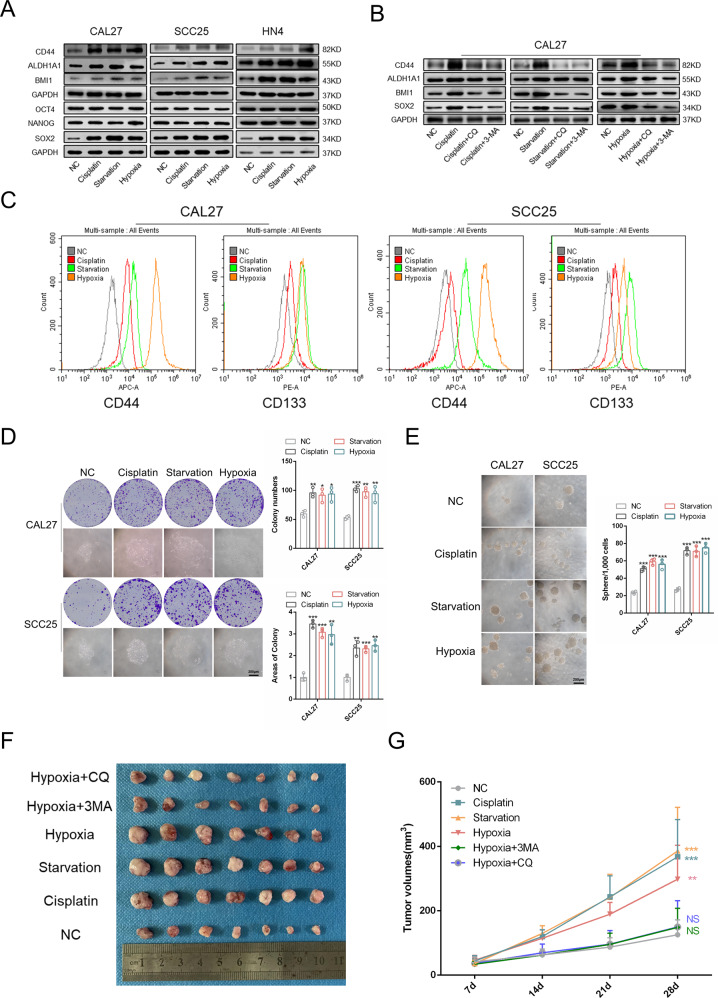
Autophagy activation is essential for the acquisition of CSC properties in cells cultured in adverse conditions. **A** Western blot was performed to determine the expression of CSCs-related markers in CAL27, SCC25, and HN4 cells when treated with cisplatin, starvation, and hypoxia. **B** Western blot was performed to determine the expression of CSCs-related markers in CAL27 cells treated with autophagy inhibitors 3-MA and CQ. **C** Flow cytometry was used to determine the expression of CD44 and CD133 in CAL27, SCC25, and HN4 cells when treated with cisplatin, starvation, and hypoxia. **D** Colony formation assay was conducted to determine the colony formation ability of CAL27 and SCC25 cells treated with cisplatin, starvation, and hypoxia. *n* = 3, Scale bars, 200 μm. **E** Sphere formation assay was carried out to determine the sphere formation ability of CAL27 and SCC25 cells treated with cisplatin, starvation, and hypoxia. *n* = 3. Scale bars, 200 μm. **F** Tumor images of cells pretreated with cisplatin, starvation, hypoxia, hypoxia + CQ, and hypoxia + 3-MA (cell dose = 3 × 10^6^). *n* = 7. **G** Tumor volumes of cells pretreated with cisplatin, starvation, hypoxia, hypoxia + CQ, and hypoxia + 3-MA. *n* = 7. Data are presented as means ± SD. *P < 0.05, **P < 0.01, and ***P < 0.001.

Previous studies have reported that cisplatin, starvation, and hypoxia are closely related to the CSC properties of tumor cells [19–22]. Therefore, we detected the stemness of cells treated with cisplatin, starvation, and hypoxia. Western blot, flow cytometry, and immunofluorescence results showed that CSC‐related markers, such as CD44, CD133, ALDH1A1, BMI1, and SOX2, were upregulated in treated cells, whereas OCT4 and NANOG were nearly unchanged (Fig. 2A, C and Supplementary Fig. 1F). CSC properties are related to self-renewal and malignancy of tumors [23]. Next, we explored the migration and invasion abilities of cancer cells using wound healing assays and Matrigel invasion assays, respectively. The results showed that tumor cells possessed stronger migration and invasion abilities when treated with cisplatin, starvation, and hypoxia (Supplementary Fig. 1A, B). We also assessed the self-renewal ability of tumor cells using a sphere formation assay and colony formation assay. The results revealed that tumor cells had elevated self-renewal ability when treated with cisplatin, starvation, and hypoxia (Fig. 2D, E). Limiting dilution analysis of CAL27 and SCC25 cells in vitro further confirmed that the treated cells had increased stemness (Supplementary Tables 1 and 2). In addition, cells pretreated with cisplatin, starvation, and hypoxia possessed stronger tumorigenicity (Fig. 2F, G). The above results demonstrated that HNSCC cells acquired stronger CSC properties in adverse conditions.

Autophagy is closely related to CSC properties in various cancers [10]. To confirm whether the elevated autophagic flux accounts for the enhanced CSC properties in adverse conditions, autophagy inhibitors 3-MA and chloroquine (CQ) were used in our studies. 3-MA and CQ markedly diminished the expression of CSC‐related markers in tumor cells treated with cisplatin, starvation, and hypoxia (Fig. 2B and Supplementary Fig. 1E). In addition, sphere formation, colony formation, and tumor formation abilities were also inhibited (Supplementary Fig. 1C, D and Fig. 2F, G). Together, these results demonstrated that HNSCC cells had stronger cell stemness under adverse conditions and that the activation of autophagy was crucial for the acquisition of CSC properties.

### Autophagy is required for the self-renewal of CSCs

**Fig. 3:**
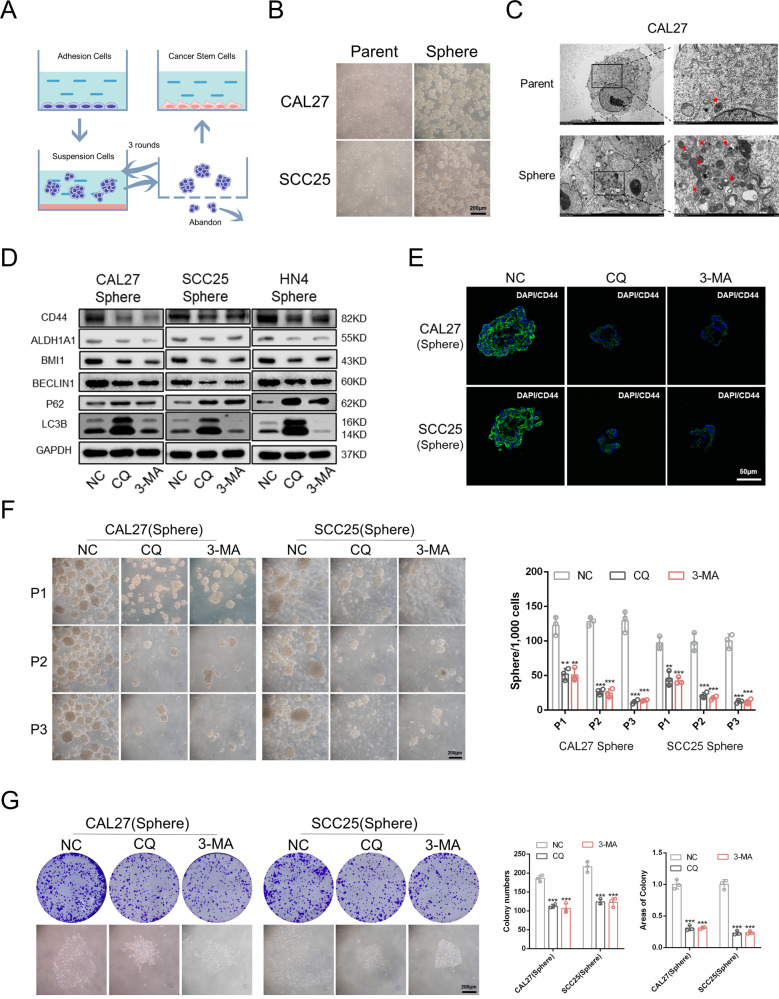
Autophagy is required for the self-renewal of CSCs. **A** Schematic diagram of the isolation of CSCs. **B** Representative morphological characteristics of primary adhesion cells and sphere cells. Scale bars, 200 μm. **C** Transmission electron microscope was conducted to observe autophagosomes in parent cells and sphere cells of CAL27. **D** Western blot was performed to determine the expression of CSCs and autophagy-related markers in sphere cells treated with autophagy inhibitors 3-MA and CQ. **E** Immunofluorescence was performed to determine CD44 expression in sphere cells when treated with 3-MA and CQ. Scale bars, 50 μm. **F** Sphere formation ability was performed to determine the sphere formation ability in successive generations of sphere cells when treated with 3-MA and CQ. *n* = 3, Scale bars, 200 μm. **G** Colony formation assay was carried out to determine the colony formation ability of sphere cells when treated with 3-MA and CQ. *n* = 3. Scale bars, 200 μm. Data are presented as means ± SD. *P < 0.05, **P < 0.01, and ***P < 0.001.

Since the activation of autophagy can promote the acquisition of CSC properties of non-CSCs, we speculated that autophagy may also be crucial for the maintenance of the CSC phenotype in HNSCC. There are several methods to isolate and identify CSCs, each with their own merits and demerits [24]. Here, we used a suspension culture assay to isolate CSCs in HNSCC (Fig. 3A). The primary adhesion cells were defined as “parent cells,” and the isolated CSCs were defined as “sphere cells” (Fig. 3B). CD44, CD133, and ALDH1A1 are crucial markers to identify CSCs from HNSCC [23, 25, 26]. We detected their expression using immunofluorescence, flow cytometry, and western blotting. The CSC‐related markers CD44, CD133, and ALDH1A1 were upregulated in sphere cells compared with parent cells (Supplementary Fig. 2A–C). In addition, the migration, invasion, sphere formation, colony formation abilities, and chemoresistance of sphere cells were stronger than those of parent cells (Supplementary Fig. 2D–G). Because the ability to form tumors in vivo is the gold standard for the identification of CSCs [24], we detected the tumor-initiating ability of sphere cells. We found that sphere cells had a higher tumor-initiating frequency (TIF) than parent cells (Supplementary Fig. 2I). Furthermore, the tumors in the sphere cell group grew more rapidly than those in the control group (Supplementary Fig. 2J). These results demonstrated that the isolated sphere cells in our experiment were indeed CSCs. Then, we explored the autophagy level in sphere cells using western blotting, immunofluorescence, and TEM. We found that Beclin1 and LC3B-II were upregulated in sphere cells, whereas p62 was decreased (Supplementary Fig. 2C). There were more autophagosomes in sphere cells (Fig. 3C and Supplementary Fig. 3A, B). These results indicated that CSCs had a higher autophagy level than non-CSCs.

Autophagy has been characterized as a requirement for the maintenance of CSCs in multiple types of cancers, such as breast cancer, lung cancer, and pancreatic cancer [27–29]. Targeting autophagy is a potential therapeutic strategy to determine the fate of CSCs [10]. In our research, the autophagy inhibitors 3-MA and CQ were used to inhibit autophagy in sphere cells. We found that the CSC-related markers CD44, ALDH1A1, and BMI1 significantly decreased when autophagy was inhibited in CAL27 and SCC25 cells (Fig. 3D). In addition, we observed lower CD44 expression and smaller spheres when autophagy was inhibited (Fig. 3E). Then, we tested the sphere formation ability in successive generations of sphere cells. We found decreases in both frequency and size of spheres when autophagy was inhibited (Fig. 3F). The colony formation ability and chemoresistance of sphere cells also decreased when treated with 3-MA and CQ (Fig. 3G and Supplementary Fig. 3C). Moreover, the migration and invasion abilities of sphere cells decreased when autophagy was inhibited (Supplementary Fig. 3D, E). Taken together, these results confirmed that the autophagy level in CSCs was higher than that in non-CSCs, and autophagy was essential for maintaining the CSC properties of HNSCC.

### Autophagy regulates CSC properties through the degradation of FOXO3

**Fig. 4:**
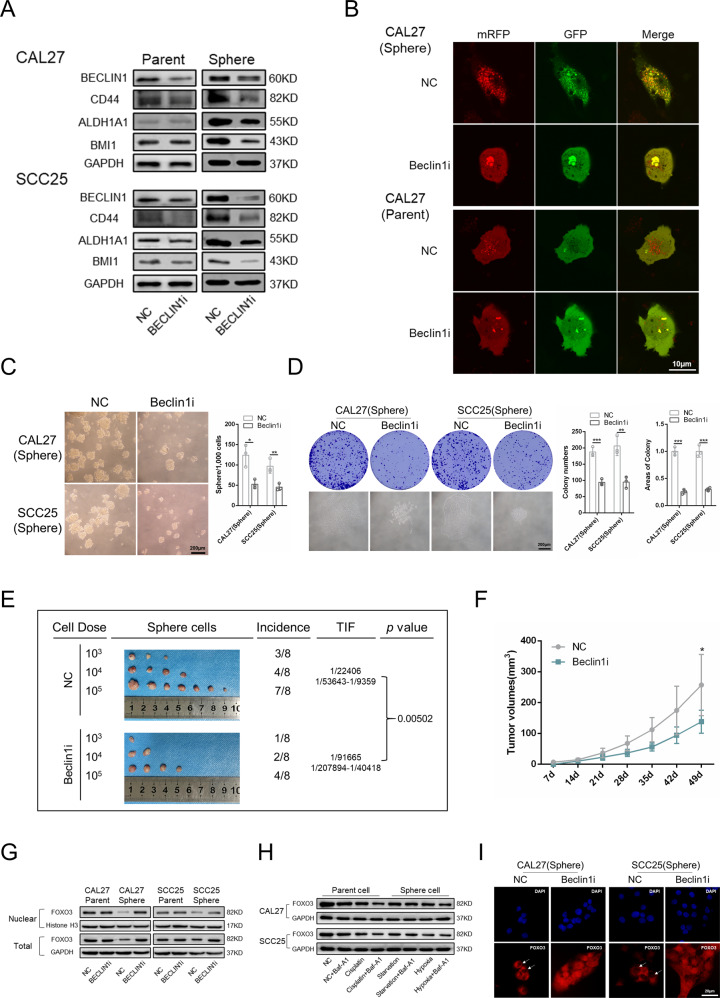
Autophagy regulates CSC properties through the degradation of FOXO3. **A** Western blot was performed to determine the expression of autophagy and CSCs-related markers in Beclin1 knockdown parent cells and sphere cells. **B** Immunofluorescence was used to observe the autophagic flux of Beclin1 knockdown parent cells and sphere cells. Scale bars, 10 μm. **C** Sphere formation ability was performed to determine the sphere formation ability of Beclin1 knockdown sphere cells. *n* = 3, Scale bars, 200 μm. **D** Colony formation assay was carried out to determine the colony formation ability of Beclin1 knockdown sphere cells. *n* = 3. Scale bars, 200 μm. **E** Beclin1 knockdown sphere cells were injected subcutaneously in the BALB/c nude mice. The tumor-initiating frequency was determined by ELDA software. *n* = 8. **F** Tumor volumes of the negative control group and Beclin1i group (cell dose = 1 × 10^5^). **G** Western blot was performed to detect the expression of FOXO3 in total cellular protein and nuclear protein of Beclin1 knockdown parent cells and sphere cells. **H** Western blot was performed to detect the expression of FOXO3 in parent cells and sphere cells when treated with cisplatin, starvation, and hypoxia. **I** Immunofluorescence was performed to observe the expression and subcellular localization of FOXO3 in Beclin1 knockdown sphere cells. Data are presented as means ± SD. *P < 0.05, **P < 0.01, and ***P < 0.001.

Beclin1 is a critical protein in the process of autophagy and is essential for the maintenance of cancer stem-like cells and tumorigenicity in vivo [30]. We knocked down the expression of Beclin1 in sphere cells using different siRNAs (Supplementary Fig. 4A). More effective Beclin1-si1 was used to construct lentiviruses (Fig. 4A). We found that autophagy was inhibited in sphere cells after Beclin1 knockdown, accompanied by decreased expressions of CSC-related proteins, which demonstrated a crucial role of autophagy in the maintenance of CSC properties (Fig. 4A, B and Supplementary Fig. 4B). However, Beclin1 knockdown had only a slight effect on the level of autophagic vesicles in the parent cells and did not markedly affect stem cell markers (Fig. 4A, B and Supplementary Fig. 4B). After Beclin1 knockdown, the sphere formation, colony formation, migration, and invasion abilities of sphere cells significantly decreased (Fig. 4C, D and Supplementary Fig. 4C, D). In addition, Beclin1 knockdown sphere cells showed lower TIF and tumor volumes (Fig. 4E, F). The survival curve acquired from the Gene Expression Profiling Interactive Analysis web tool (http://gepia.cancer-pku.cn/) revealed that high expression of Beclin1 was correlated with poor survival of HNSCC (Supplementary Fig. 4I) [31].

Recently, an interesting mechanism has been uncovered. FOXO3, which is an autophagy-regulating transcription factor, is itself degraded by basal autophagy [17]. Considering the tumor-suppressive roles of FOXO3 in various cancers [13], we hypothesized that autophagy could regulate CSC properties by degrading FOXO3. Western blot results showed that the expression of FOXO3 changed significantly in sphere cells but only changed slightly in parent cells when Beclin1 was knocked down (Fig. 4G). In contrast, treatment with cisplatin, starvation, and hypoxia markedly affected FOXO3 expression in parent cells but had a slight effect on sphere cells (Fig. 4H). We hypothesized that the reason was the different basic autophagy levels of parent cells and sphere cells. Neither Beclin1 knockdown significantly decreased the autophagic flux of parent cells, nor did treatment with cisplatin, starvation, or hypoxia substantially enhance the autophagic flux of sphere cells. In addition, the mRNA expression of FOXO3 was nearly unchanged in Beclin1 knockdown sphere cells, which demonstrated that FOXO3 was regulated by autophagy at the protein level but not at the mRNA level (Supplementary Fig. 4K). FOXO3 functions by binding to specific DNA fragments and changes the pattern of transcriptional activity of target genes in the cell nucleus [32]. We found that FOXO3 was enriched in the cell nucleus when Beclin1 was knocked down (Fig. 4G, I), indicating that FOXO3 could function better when autophagy was inhibited. Overall, these results suggested that autophagy probably maintained CSC properties through the degradation of FOXO3.

### FOXO3 regulates the CSC phenotype through transcriptional inhibition of SOX2

**Fig. 5:**
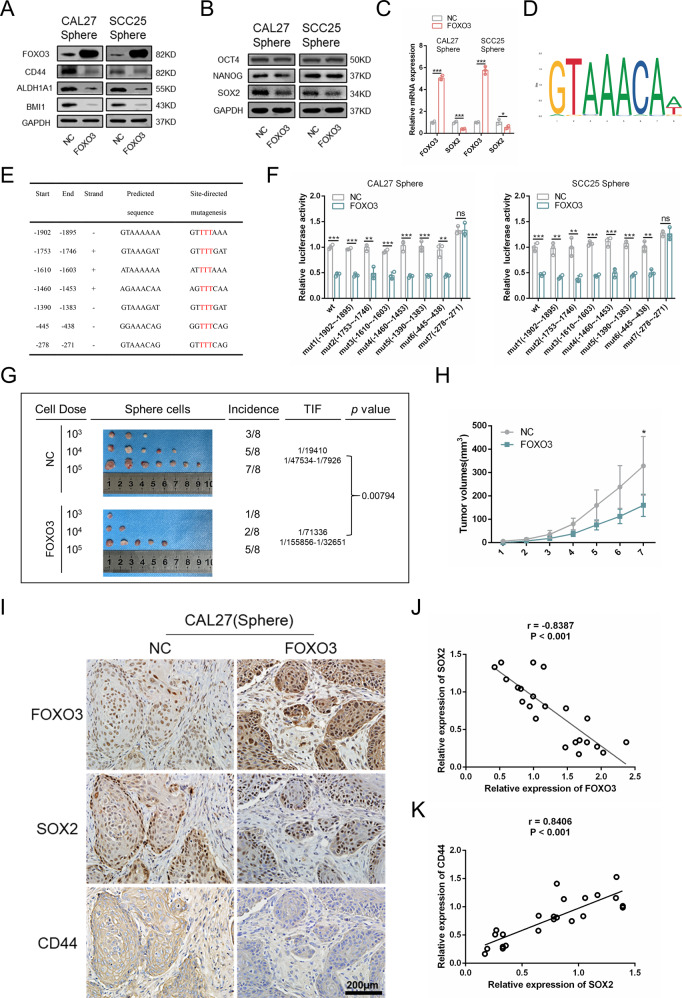
FOXO3 regulates the CSC phenotype through transcriptional inhibition of SOX2. **A** Western blot was performed to detect the expression of CD44, ALDH1A1, and BMI1 in FOXO3 overexpression sphere cells. **B** Western blot analysis of the expression of OCT4, NANOG, and SOX2 in FOXO3 overexpression sphere cells. **C** Real‐time PCR was conducted to detect mRNA expression of SOX2 in FOXO3 overexpression sphere cells. **D** The binding motif of FOXO3. **E** The potential binding sites of FOXO3 in SOX2 promoter region. **F** Dual-luciferase reporter assay was performed to verify the binging sequence of FOXO3 in the SOX2 promoter region. **G** FOXO3 overexpression sphere cells were injected subcutaneously in the BALB/c nude mice. The tumor-initiating frequency was determined by ELDA software. *n* = 8. **H** Tumor volumes of negative control group and FOXO3 group (cell dose = 1 × 10^5^). **I** The expression of FOXO3, SOX2, and CD44 in xenograft tumors was detected via immunohistochemistry. Scale bars, 200 μm. **J** The correction analysis between FOXO3 and SOX2 expression in xenograft tumors was determined via Spearman rank correlation analysis. *n* = 23. **K** The correction analysis between SOX2 and CD44 expression in xenograft tumors was determined via Spearman rank correlation analysis. *n* = 23. Data are presented as means ± SD. *P < 0.05, **P < 0.01, and ***P < 0.001.

FOXO3 is commonly regarded as a tumor-suppressive gene in cancer [32]. We found that low expression of FOXO3 was correlated with poor survival of HNSCC (Supplementary Fig. 4J). Recent studies have demonstrated that FOXO3 is associated with the properties of CSCs [16, 33–35]. To further investigate the mechanisms by which FOXO3 regulated the stemness of HNSCC, FOXO3-overexpressing cell lines were constructed. We found that CSC-related markers, such as CD44, ALDH1A1, and BMI1, decreased significantly when FOXO3 was overexpressed (Fig. 5A). The sphere formation, colony formation, migration, and invasion abilities of sphere cells also decreased when FOXO3 was overexpressed (Supplementary Fig. 4E–H). In addition, FOXO3-overexpressing cells showed lower TIF and tumor volumes in vivo (Fig. 5G, H). SOX2, OCT4, and NANOG are critical transcription factors that can regulate the properties of CSCs [36, 37]. We found that SOX2 was significantly decreased in FOXO3-overexpressing cells, whereas OCT4 and NANOG were nearly unchanged (Fig. 5B, C), indicating that SOX2 might be a target of FOXO3. Next, we searched the motif of the transcription factor FOXO3 and found seven binding sites of FOXO3 in the promoter region (between −2000 and 0) of SOX2 through results acquired from JASPAR (http://jaspar.genereg.net) (Fig. 5D, E) [38]. To verify whether FOXO3 could directly regulate the promoter region of SOX2, a dual-luciferase reporter assay was performed. Different SOX2 promoter mutants were cloned into the pGL3-basic luciferase plasmid. Meanwhile, the internal control phRL-TK plasmid was cotransfected into CAL27 and SCC25 cells. We found that FOXO3 could bind to the “GTAAACAG” sequence of the SOX2 promoter and inhibit its transcriptional activity (Fig. 5F). Immunohistochemical staining of tumors showed that the FOXO3 overexpression group had lower expression of SOX2 and CD44 (Fig. 5I). Moreover, correlation analysis revealed that the expression of FOXO3 was negatively correlated with the expression of SOX2, and the expression of SOX2 was positively correlated with the expression of CD44 (Fig. 5J, K).

**Fig. 6:**
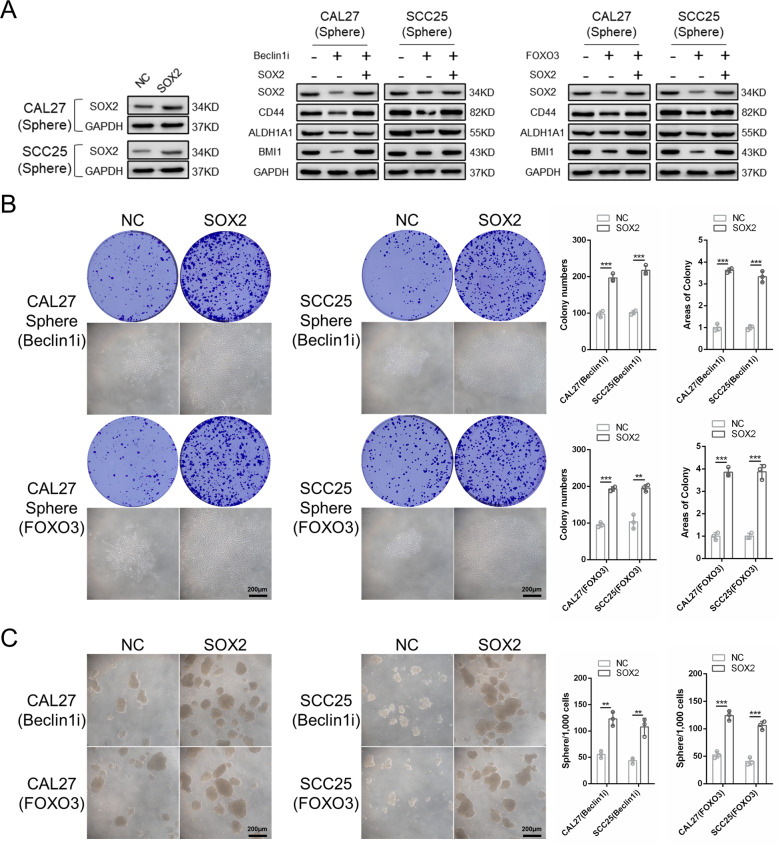
Loss of SOX2 is indeed the reason for loss of stemness in Beclin1 knockdown and FOXO3 overexpression sphere cells. **A** Western blot analysis of the CSCs-related markers in SOX2 overexpression sphere cells. **B** Colony formation assay was carried out to determine the colony formation ability of SOX2 overexpression sphere cells. *n* = 3. Scale bars, 200 μm. **C** Sphere formation ability was performed to determine the sphere formation ability of SOX2 overexpression sphere cells. *n* = 3, Scale bars, 200 μm. Data are presented as means ± SD. *P < 0.05, **P < 0.01, and ***P < 0.001.

To further confirm that the loss of SOX2 was indeed the key reason for the loss of stemness. We overexpressed SOX2 in Beclin1 knockdown and FOXO3 overexpression sphere cells. We found that CSC-related markers were significantly enhanced when SOX2 was overexpressed (Fig. 6A). In addition, both sphere formation and colony formation abilities were ameliorated by SOX2 overexpression (Fig. 6B, C). Altogether, these results demonstrated that FOXO3 regulated the CSC phenotype through the transcriptional inhibition of SOX2.

## Discussion

**Fig. 7:**
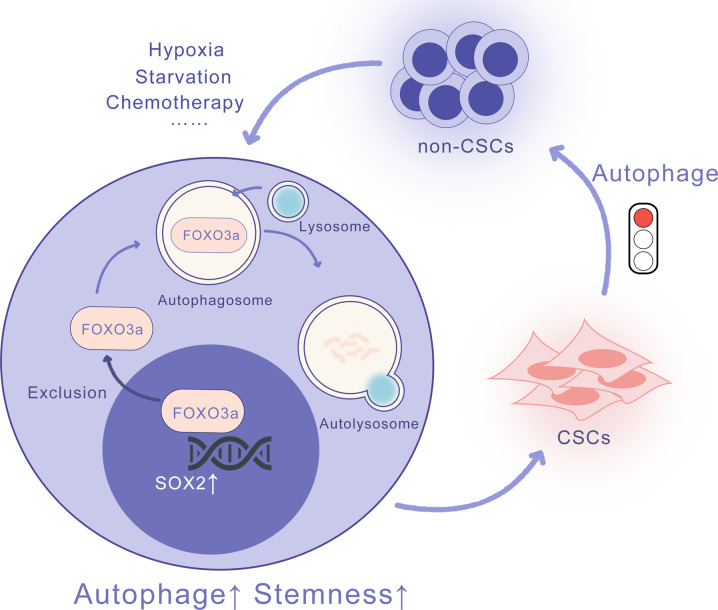
Schematic diagram of the mechanisms of autophagy in regulating CSCs phenotype of HNSCC. In adverse conditions, such as chemotherapy, starvation, and hypoxia, the autophagy level increased in HNSCC. Then, the elevated autophagy degrades FOXO3 in the cytoplasm and promotes its nuclear exclusion. The decreased FOXO3 in the nucleus alleviates its transcriptional inhibition of SOX2, thus enhancing CSCs properties of HNSCC. Conversely, when autophagy is inhibited in CSCs, FOXO3 accumulates in the nucleus and inhibited the transcription of SOX2, thus promoting CSCs transformed into non-CSCs.

Cancer is a dynamic disease in which the interconversion of non-CSCs and CSCs exists. CSCs possess different biological properties and therapeutic sensitivities than non-CSCs and contribute to inferior clinical outcomes of patients [37, 39]. The study and search for effective targets for CSCs are current trends in curing cancers. In our research, we found that autophagy and stemness of HNSCC increased significantly when treated with cisplatin, starvation, and hypoxia, and the activation of autophagy was crucial for the acquisition of CSC properties. In addition, the isolated CSCs had a higher autophagic level than primary adhesion cells. The inhibition of autophagy dramatically reduced the stemness of CSCs. Furthermore, we found that autophagy enhanced CSC properties through the degradation of FOXO3. FOXO3, a transcription factor that can inhibit the transcription of SOX2, has tumor-suppressive functions in CSCs. Our research clarified the role and intrinsic mechanism of autophagy in regulating CSC properties and proposed autophagy as a potential therapeutic target for HNSCC (Fig. 7).

Autophagy, a catabolic process for the degradation of unnecessary or dysfunctional cellular components, plays complicated roles in tumor suppression and promotion [9]. In the initial stage of tumors, autophagy usually functions as a tumor suppressor by mechanically eliminating oxidative stress, damaged organelles, and genomic instability. Takamura et al. found that autophagy-deficient hepatocytes had higher risks of developing liver adenomas, as these cells showed obvious oxidative stress and genomic damage responses [40]. However, in established tumors, autophagy can function as a tumor promoter by providing energy for tumor growth and enhancing resistance to unfavorable survival conditions. Yang et al. found that autophagy played a cytoprotective role in HNSCC and that blocking autophagy promoted the therapeutic effect of interferon-alpha [41]. In our research, we found that autophagy in HNSCC was elevated after treatment with cisplatin, starvation, and hypoxia. These results were consistent with previous studies showing that autophagy could respond to adverse conditions and maintain cell homeostasis. In addition, we found that elevated autophagic flux was accompanied by enhanced cell stemness, and the autophagy inhibitors 3-MA and CQ attenuated cisplatin-, starvation-, and hypoxia-induced stemness, indicating that autophagy was required for the acquisition of CSC properties.

CSCs are a subtype of cells with symmetrical self‐renewal and asymmetrical differentiation abilities and have been shown to be highly resistant to chemoradiotherapy [24]. Previous studies have found that autophagy is upregulated in various CSCs, including breast CSCs, ovarian CSCs, glioblastoma CSCs, and pancreatic CSCs [28, 30, 42, 43]. Since autophagy is a vital biological process for maintaining cell homeostasis in unfavorable circumstances, the stronger chemoradiotherapy resistance of CSCs can be inferred partly due to elevated autophagy levels. In our research, we found that isolated CSCs possessed higher autophagic flux and chemoresistance. The inhibition of autophagy using 3-MA and CQ significantly decreased the chemoresistance of CSCs. These results validated our hypothesis and were consistent with past research. In addition, we found that the sphere formation, colony formation, migration, and invasion abilities of CSCs decreased when autophagy was inhibited. Knockdown of Beclin1, an autophagy-related gene, impaired the self-renewal potential and reduced the TIF of CSCs. Our results demonstrated that autophagy was essential for the maintenance of the CSC phenotype in HNSCC.

FOXO3 can mediate various biological processes by regulating the transcription of its target genes [13]. Recently, Fitzwalter et al. found that FOXO3, an autophagy-regulating transcription factor, was itself degraded by basic autophagy. The inhibition of autophagy resulted in the accumulation of FOXO3 in the nucleus, which then promoted the transcription of proapoptotic genes [17]. Their research linked autophagy and apoptosis through a single transcription factor and revealed for the first time that FOXO3 was a substrate for autophagy. In our research, when autophagy was inhibited, FOXO3 increased significantly and translocated into the nucleus. The inhibition of autophagy enhanced the transcriptional activity of FOXO3. Our study demonstrated that FOXO3 remained a substrate for autophagy in CSCs. FOXO3 is generally considered an anti-oncogene, and its inactivation is associated with the occurrence and development of various cancers [13]. Moreover, FOXO3 is related to CSC properties in some kinds of tumors [15, 16, 33–35]. For example, Liu et al. reported that DNMT1-mediated downregulation of FOXO3a promoted CSC properties and tumorigenesis in breast cancer; FOXO3a was functionally correlated with the inhibition of FOXM1/SOX2 signaling [15]. In our research, we found that FOXO3 regulated CSC properties through the transcriptional inhibition of SOX2, which is a key transcription factor for the maintenance of CSCs. Sphere formation, colony formation, migration, invasion abilities, and TIF decreased in CSCs when FOXO3 was overexpressed. The expression of FOXO3 was negatively correlated with the stemness of HNSCC. Our research indicated the complementary roles of FOXO3 in different types of cancers.

In conclusion, our findings revealed that autophagy is an important biological process in regulating CSC properties. The FOXO3/SOX2 axis is the key signaling pathway involved in the autophagy-regulated CSC phenotype. Autophagy may have potential as a therapeutic target to eliminate CSCs in HNSCC.

## Materials and methods

### Cell lines and culture

CAL27, SCC25, and HN4 cells were obtained from the China Center for Type Culture Collection (Shanghai, China) and passaged fewer than 6 months after purchase. All cell lines were confirmed to be mycoplasma-free and authenticated by short tandem repeat loci profiling. CAL27 and HN4 cells were cultivated in DMEM. SCC25 cells were cultivated in DMEM/F-12. Antibiotics and 10% FBS were added to the medium as supplements. Cells were cultured at 37 °C in a humidified atmosphere containing 5% CO_2_.

### Isolation of CSCs

Single-cell suspensions (10^3^ cells/well) were cultured in ultralow attachment 6-well plates with CSC-specific medium. Every 7 days, spheres were collected using a 70-μm cell strainer and resuspended in single cells using tryptase. Three rounds later, the acquired cells were identified as CSCs.

### RNA interference and lentiviral transfection

Control siRNAs (Beclin1-siNC, 5ʹ-UUCUCCGAACGUGUCACGUTT-3ʹ) and two different siRNAs (Beclin1-si1, 5ʹ-GAGGAUGACAGUGAACAGUUA-3ʹ; Beclin1-si2, 5ʹ-GCUCAGUAUCAGAGAGAAUTT-3ʹ) targeting Beclin1 were purchased from Hanbio Technology (Shanghai, China). More effective Beclin1-si1 was used to construct lentiviruses. The recombinant lentivirus was purchased from GeneChem (Shanghai, China). A lentivirus named Beclin1i was used to interfere with the expression of Beclin1. Lentiviruses named FOXO3 and SOX2 were used to increase the expression of FOXO3 and SOX2 separately. Cells were transfected with lentivirus for 72 h and screened with puromycin (1 μg/ml) for 1 week to establish stably transfected cell lines. mRFP-EGFP-LC3 adenovirus was purchased from HanBio Technology. In the merged images, autophagosomes are shown as yellow puncta, and autolysosomes are shown as red puncta. Autophagic flux is increased when both yellow and red puncta are increased in cells.

### Transmission electron microscopy (TEM) analysis

The primary adhesion cells and sphere cells were harvested and fixed in glutaraldehyde for 2 h. Then, the cells were postfixed in 1% osmic acid for 2 h and dehydrated using graded ethanol (50–100%). Subsequently, the cells were embedded in Spurr’s epoxy resin and cut into ultrathin sections (60 μm). Finally, the sections were stained with uranyl acetate and lead citrate. TEM (HT7700, Hitachi, Japan) was used to acquire images of autophagosomes in cells.

### Wound healing assay and Matrigel invasion assay

The experiments proceeded as previously described [44]. A wound healing assay was used to assess cell migration. A Matrigel invasion assay was used to assess cell invasion.

### Colony formation assay and sphere formation assay

The experiments were carried out following the protocol of our previous study [44]. We counted the number of colonies under a microscope and evaluated the areas of colonies using Image-Pro Plus. A colony formation assay was used to assess the colony-forming efficiency of cells. We tested the cell sphere formation ability by growing spheroids from spheroid-derived cells for successive generations. A sphere formation assay was used to assess the sphere formation ability of cells.

### Western blotting and real‐time PCR assay

We conducted the experiments as previously described [44]. Beclin1 (11306-1-AP), P62 (18420-1-AP), CD44 (15675-1-AP), ALDH1A1 (15910-1-AP), BMI1 (10832-1-AP), OCT4 (11263-1-AP), and GAPDH (60004-1-Ig) antibodies were purchased from Proteintech (Wuhan, China). Antibodies against LC3B (3868S), FOXO3 (12829S), and SOX2 (3579S) were purchased from CST (Danvers, MA, USA). The primers were designed and synthesized by Sangon Biotech (Shanghai, China). The primer sequences were as follows: Beclin1 (F: 5ʹ-CCATGCAGGTGAGCTTCGT‐3ʹ, R: 5ʹ‐GAATCTGCGAGAGACACCATC‐3ʹ), FOXO3 (F: 5ʹ-CGGACAAACGGCTCACTCT‐3ʹ, R: 5ʹ‐GGACCCGCATGAATCGACTAT‐3ʹ), SOX2 (F: 5ʹ-TGGACAGTTACGCGCACAT‐3ʹ, R: 5ʹ‐CGAGTAGGACATGCTGTAGGT‐3ʹ), and GAPDH (F: 5ʹ‐GGAGCGAGATCCCTCCAAAAT‐3ʹ, R: 5ʹ-GGCTGTTGTCATACTTCTCATGG‐3ʹ).

### Immunofluorescence and immunohistochemistry

For cell immunofluorescence, adherent cells were seeded on a 24-well plate with prepared cell climbing glasses. Sphere cells were collected and embedded in optimum cutting temperature compound (SAKURA, Torrance, CA, USA) and sliced at 6 μm. For tissue immunohistochemistry, xenograft tumors were embedded in paraffin and cut at 5 μm. The experiments were conducted following the protocol of our previous study [44]. Fluorescence microscopy (Biozero BZ‐8000; Keyence, Osaka, Japan) and confocal microscopy (Andor Revolution XD; Andor Belfast, United Kingdom) were utilized to acquire images of stained cells.

### Flow cytometry

Single-cell suspensions were prepared and washed in PBS containing 1% BSA. Next, the cells were incubated with PE-conjugated anti-CD133 (566593, BD Pharmingen, San Diego, CA, USA) and APC-conjugated anti-CD44 (559942, BD Pharmingen) antibodies for 15 min at 4 °C. Labeled cells were washed again and suspended in PBS. Flow cytometry (BD Biotechnology) was used to detect the expression of CD133 and CD44. The apoptosis experiment was carried out following the protocol of our previous study [44].

### Luciferase assay

The phRL-TK plasmid, pGL3-basic luciferase plasmid, and pGL3 luciferase plasmid containing different SOX2 promoters were purchased from MiaoLing (Wuhan, China). CAL27 and SCC25 cells were cultured on a 48-well plate and transfected with 0.2 μg reporter plasmid and 0.02 μg phRL-TK plasmid using Lipofectamine 3000 (Invitrogen, Carlsbad, CA, USA). Forty-eight hours later, we measured luciferase activity using the Dual-Luciferase Reporter Assay Kit (Promega, Madison, MI, USA). SOX2 transcriptional activity was in direct proportion to firefly luciferase/Renilla luciferase (fRLU/rRLU) activity.

### Mouse xenografts

The mouse experiment was approved by the Ethics Committee of School and Hospital of Stomatology, Wuhan University (Approval number: 2016LUNSHENZI60). Female BALB/c nude mice (age 6 weeks) were purchased from Beijing Vital River Laboratory Animal Technology Co., Ltd. (Beijing, China). The mice were randomly divided into different groups. For tumor formation ability analysis, 3 × 10^6^ CAL27 cells (*n* = 7) were resuspended in 50 μl PBS mixed with 50 μl Matrigel and injected subcutaneously into the mice. For tumor-initiating capacity analysis, 10^3^, 10^4^, and 10^5^ CAL27 cells (*n* = 8) were resuspended in 50 μl PBS mixed with 50 μl Matrigel and injected subcutaneously into the mice. We measured the size of the tumor blindly every 3 days and calculated tumor volumes using the formula (width^2^ × length)/2. The frequency of tumor-initiating cells was examined using Extreme Limiting Dilution Analysis software (http://bioinf.wehi.edu.au/software/elda/index.html) [45]. No animals were excluded from the analysis.

### In vitro limiting dilution assay

This experiment was conducted following the protocol of Bioprotocol [46].

### Statistical analysis

No statistical method was used to choose a sample size. The variance within each group was similar. Student’s *t*-test was used to analyze data between two groups. One‐way analysis of variance was used to analyze data between multiple groups. Spearman rank correlation analysis was used to analyze correlations between the data of two groups. All results were calculated in at least three independent experiments and are presented as the means ± SEM. A two-tailed value of *P* < 0.05 was defined as statistically significant (*P < 0.05, **P < 0.01, and ***P < 0.001).

## Supplementary information

Supplementary Figures

Supplementary Table 1

Supplementary Table 2
