# Supplementary material for: Autophagy regulates the cancer stem cell phenotype of head and neck squamous cell carcinoma through the noncanonical FOXO3/SOX2 axis
Source: Oncogene. 2021 Nov 19;41(5):634–46. doi: 10.1038/s41388-021-02115-7 (PMC8799462; doi:10.1038/s41388-021-02115-7)
Supplement: Supplementary file 1 — Supplementary Figures [file 41388_2021_2115_MOESM1_ESM.docx]

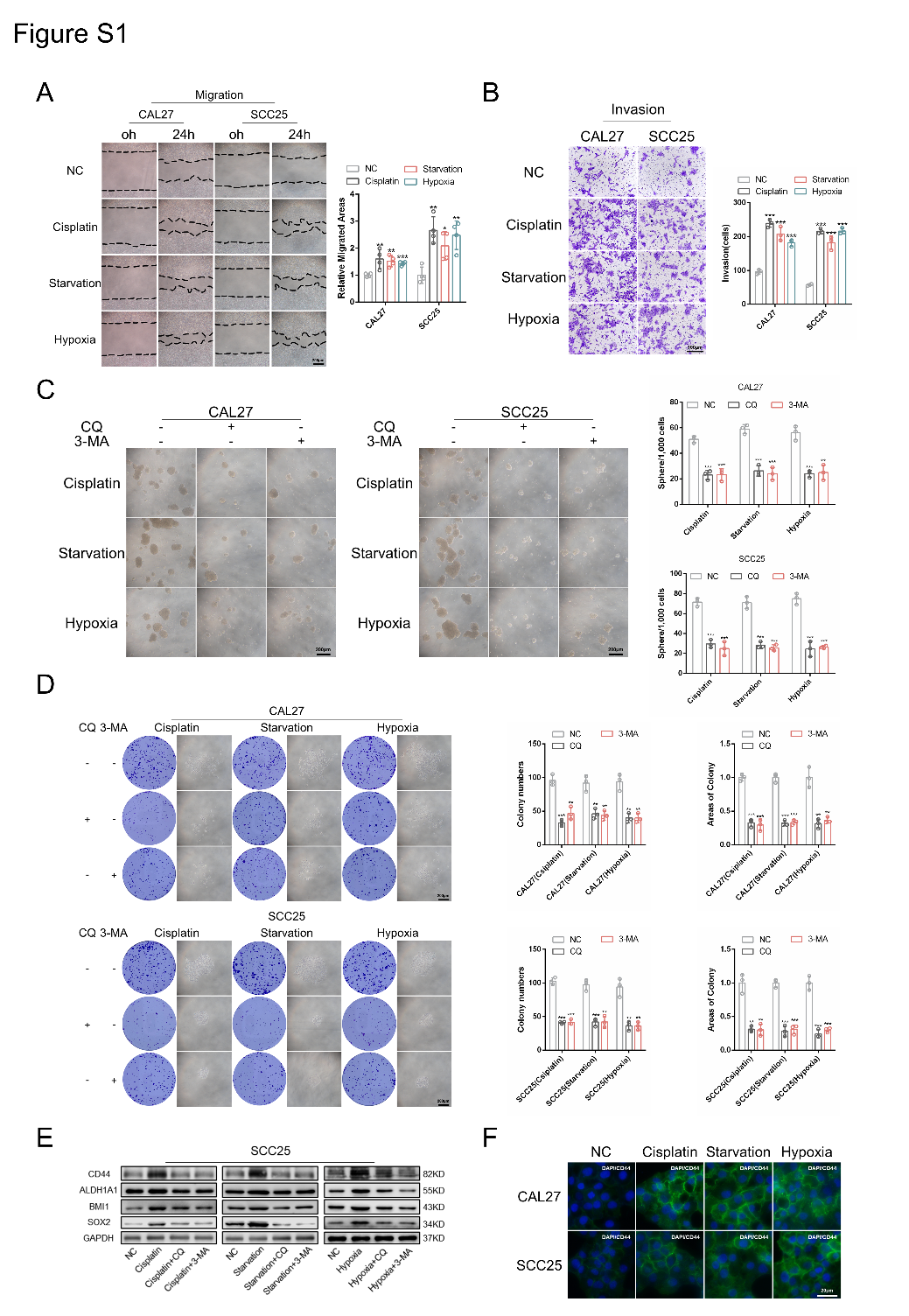


Figure S1. a Wound healing assay was performed to determine the migration ability of CAL27 and SCC25 cells treated with cisplatin, starvation, and hypoxia. n = 4. Scale bars, 200 μm. b Matrigel invasion assay was performed to determine the invasion ability of CAL27 and SCC25 cells treated with cisplatin, starvation, and hypoxia. n = 3. Scale bars, 100 μm. c Sphere formation assay was carried out to determine the sphere formation ability of cells treated with autophagy inhibitors 3-MA and CQ. n = 3. Scale bars, 200 μm. d Colony formation assay was conducted to determine the colony formation ability of cells treated with autophagy inhibitors 3-MA and CQ. n = 3, Scale bars, 200 μm. e Western blot was performed to determine the expression of CSCs-related markers in SCC25 cells treated with autophagy inhibitors 3-MA and CQ. f Immunofluorescence was performed to determine the CD44 expression in CAL27 and SCC25 cells when treated with cisplatin, starvation, and hypoxia. Scale bars, 20 μm. Data are presented as means ± SD. * P＜0.05, ** P＜0.01, and *** P＜0.001.


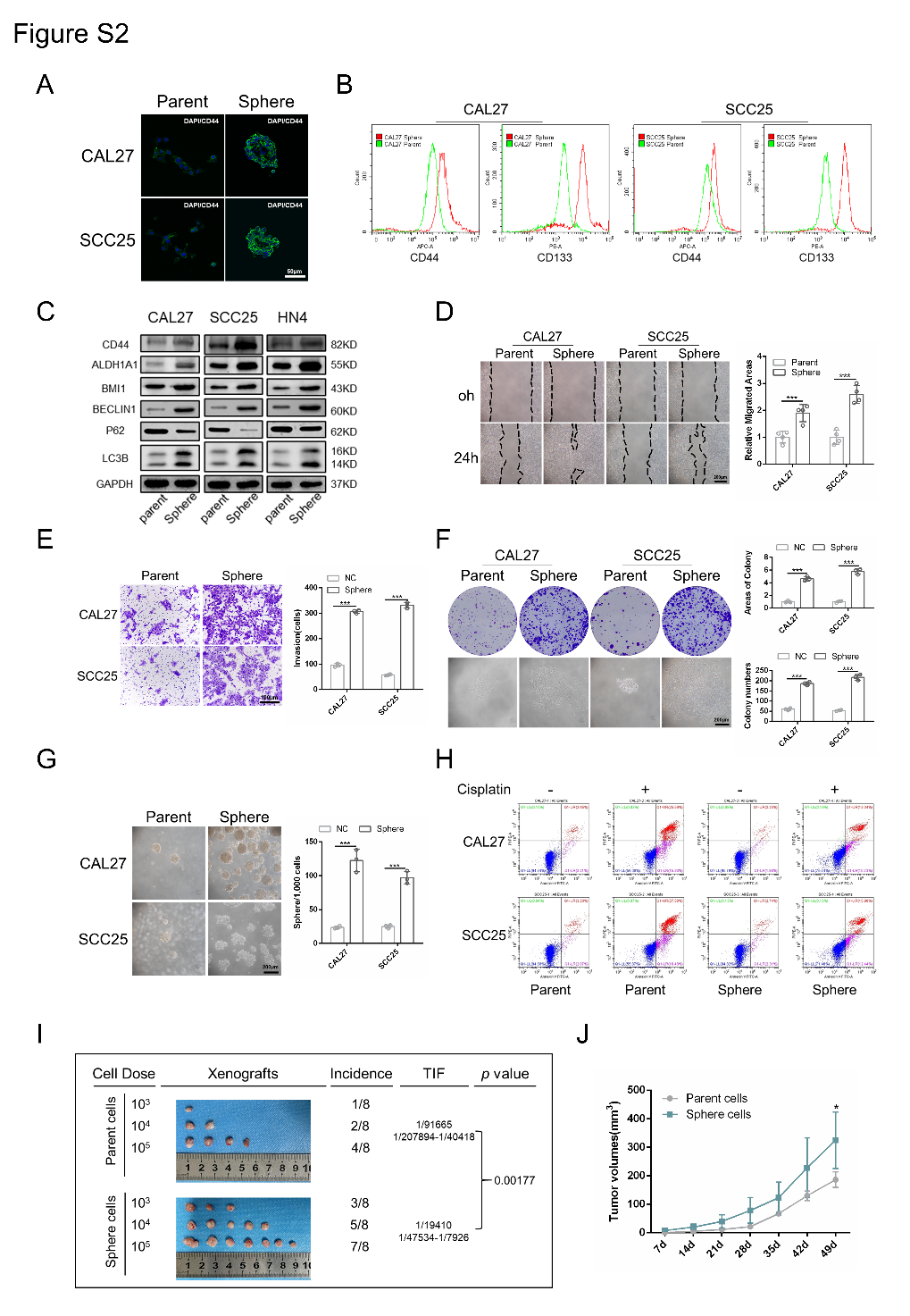


Figure S2. a Immunofluorescence was performed to determine the CD44 expression of parent cells and sphere cells. Scale bars, 50 μm. b The expression of CD44 and CD133 of parent cells and sphere cells were detected by flow cytometry. c Western blot was performed to detect the CSCs and autophagy-related markers of parent cells and sphere cells. d Wound healing assay was performed to determine the migration ability of parent cells and sphere cells. n = 4. Scale bars, 200 μm. e Matrigel invasion assay was performed to determine the invasion ability of parent cells and sphere cells. n = 3. Scale bars, 100 μm. f Colony formation assay was conducted to determine the colony formation ability of parent cells and sphere cells. n = 3, Scale bars, 200 μm. g Sphere formation assay was carried out to determine the sphere formation ability of parent cells and sphere cells. n = 3. Scale bars, 200 μm. h Flow cytometry was performed to detect the chemoresistance of parent cells and sphere cells. i Parent cells and sphere cells were injected subcutaneously in the BALB/c nude mice. The tumor-initiating frequency was determined by ELDA software. n = 8. j Tumor volumes of parent cells group and sphere cells group (cell dose = 1×105). Data are presented as means ± SD. * P＜0.05, ** P＜0.01, and *** P＜0.001.


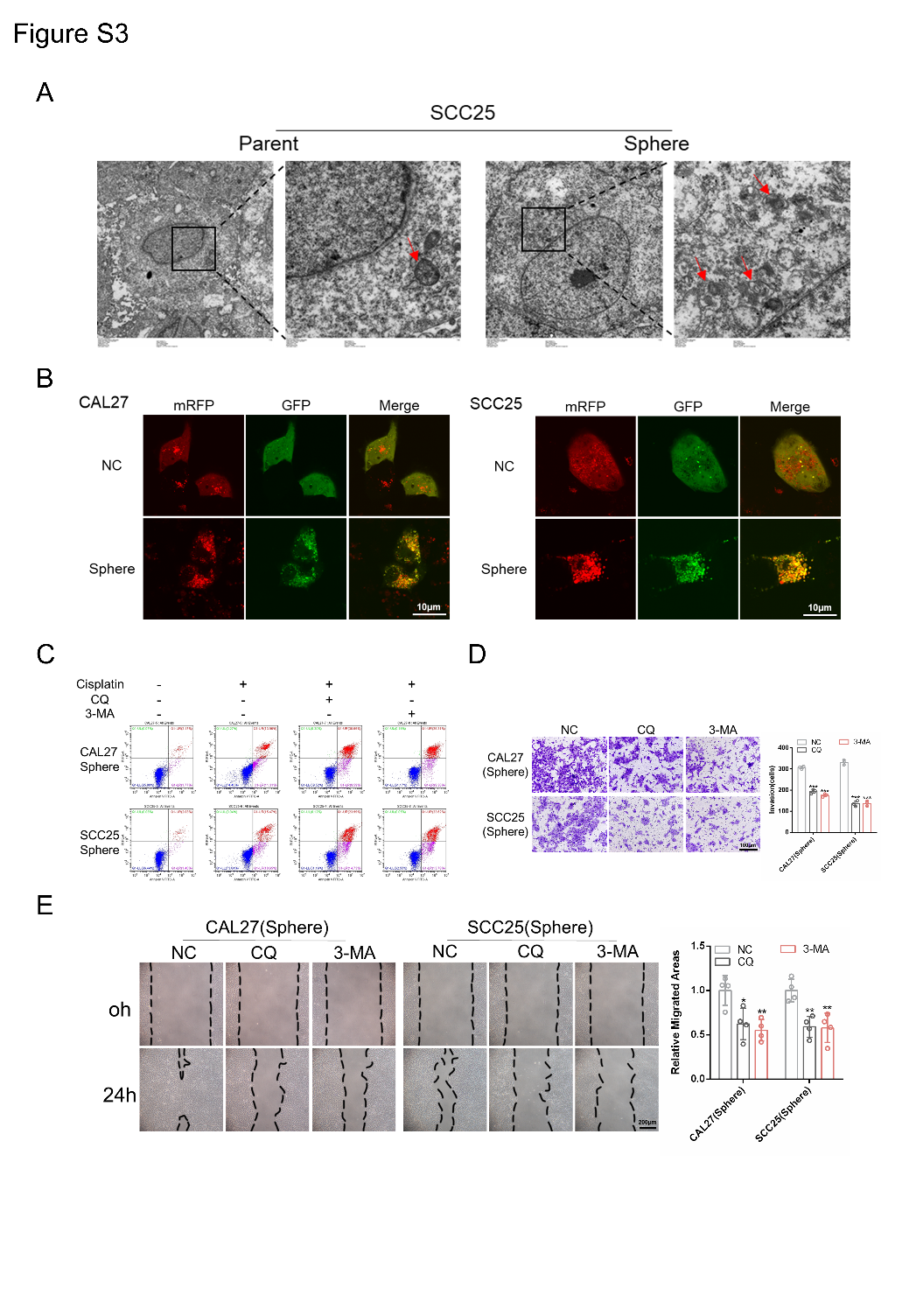


Figure S3. a Transmission electron microscope was conducted to observe autophagosomes in parent cells and sphere cells of SCC25. b Immunofluorescence was used to observe the autophagic flux of parent cells and sphere cells. Scale bars, 10 μm. c Flow cytometry was performed to detect the chemoresistance of sphere cells when treated with 3-MA and CQ. d Matrigel invasion assay was performed to determine the invasion ability of sphere cells when treated with 3-MA and CQ. n = 3. Scale bars, 100 μm. e Wound healing assay was performed to determine the migration ability of sphere cells when treated with 3-MA and CQ. n = 4. Scale bars, 200 μm. Data are presented as means ± SD. * P＜0.05, ** P＜0.01, and *** P＜0.001.


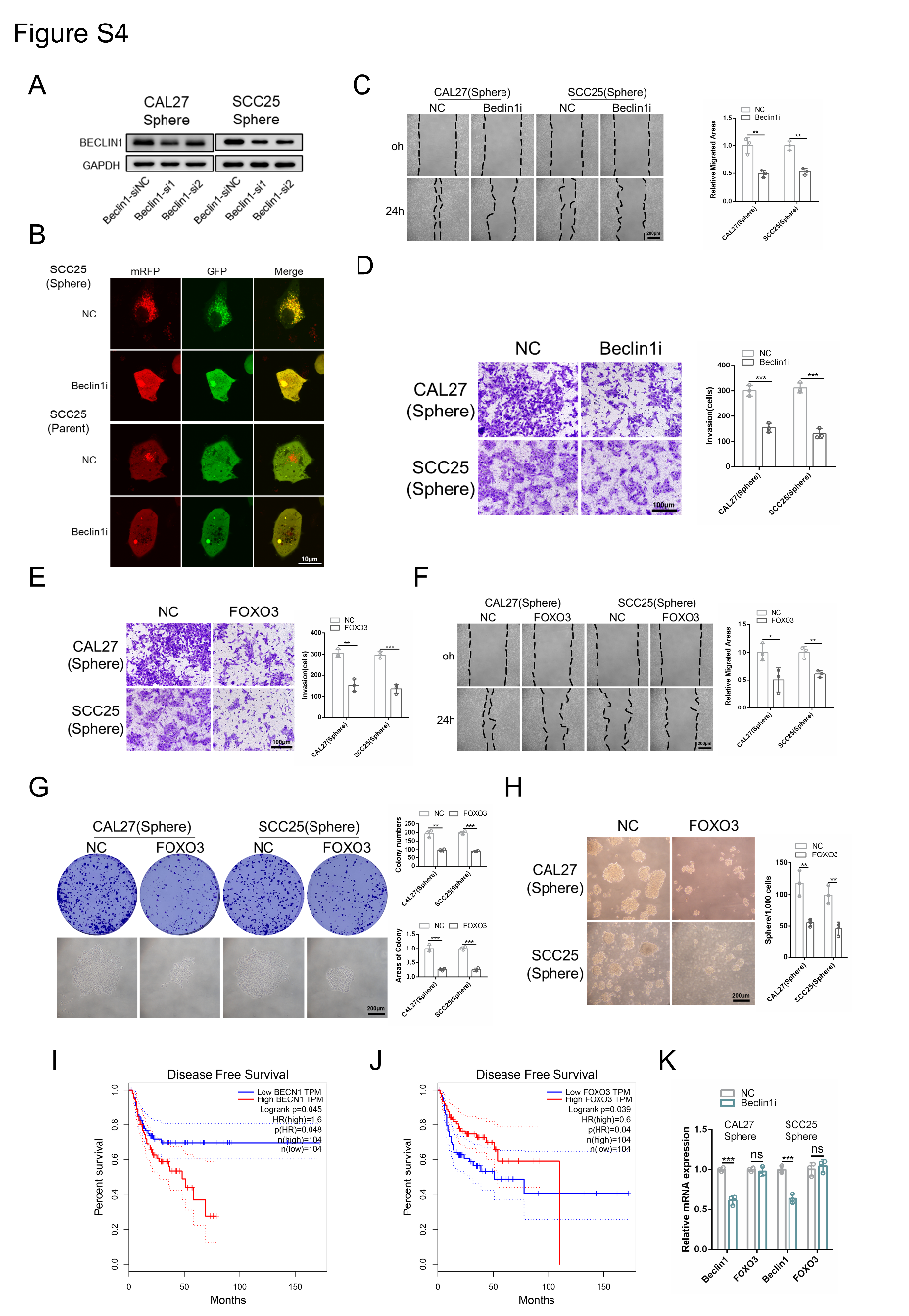


Figure S4. a Western blot was performed to determine the Beclin1 expression of CAL27 and SCC25 cells. b Immunofluorescence was used to observe the autophagic flux of Beclin1 knockdown parent cells and sphere cells. Scale bars, 10 μm. c Wound healing assay was performed to determine the migration ability of Beclin1 knockdown sphere cells. n = 4. Scale bars, 200 μm. d Matrigel invasion assay was performed to determine the invasion ability of Beclin1 knockdown sphere cells. n = 3. Scale bars, 100 μm. e Matrigel invasion assay was performed to determine the invasion ability of FOXO3 overexpression sphere cells. n = 3. Scale bars, 100 μm. f Wound healing assay was performed to determine the migration ability of FOXO3 overexpression sphere cells. n = 4. Scale bars, 200 μm. g Colony formation assay was carried out to determine the colony formation ability of FOXO3 overexpression sphere cells. n = 3. Scale bars, 200 μm. h Sphere formation ability was performed to determine the sphere formation ability of FOXO3 overexpression sphere cells. n = 3. Scale bars, 200 μm. i The disease-free survival curve of HNSCC acquired from GEPIA according to Beclin1 expression. j The disease-free survival curve of HNSCC acquired from GEPIA according to FOXO3 expression. k Real‐time PCR was conducted to detect mRNA expression of FOXO3 in Beclin1 knockdown sphere cells. n = 3. Data are presented as means ± SD. * P＜0.05, ** P＜0.01, and *** P＜0.001.
