# Supplementary material for: Autophagy regulates the cancer stem cell phenotype of head and neck squamous cell carcinoma through the noncanonical FOXO3/SOX2 axis
Source: Oncogene. 2021 Nov 19;41(5):634–46. doi: 10.1038/s41388-021-02115-7 (PMC8799462; doi:10.1038/s41388-021-02115-7)
Supplement: Supplementary file 2 — Supplementary Table 1 [file 41388_2021_2115_MOESM2_ESM.docx]

**Table S1. Limiting Dilution Analysis of CAL27 in vitro**

| **Group** | **Cell Dose** | **Incidence** | **Frequency** | **P value (vs. NC)** |
| --- | --- | --- | --- | --- |
| NC | 100 | 24/24 | 1/20.64(1/33.53-1/12.78) |  |
|  | 10 | 9/24 |  |  |
| Cisplatin  Starvation  Hypoxia | 1  100  10  1  100  10  1  100  10  1 | 2/48  24/24  16/24  19/48  24/24  15/24  23/48  24/24  18/24  20/48 | 1/5.46(1/7.90-1/3.83)  1/5.15(1/7.43-1/3.63)  1/4.57(1/6.55-1/3.24) | 6.3e-06  1.41e-06  1.85e-07 |
