# Supplementary material for: Autophagy regulates the cancer stem cell phenotype of head and neck squamous cell carcinoma through the noncanonical FOXO3/SOX2 axis
Source: Oncogene. 2021 Nov 19;41(5):634–46. doi: 10.1038/s41388-021-02115-7 (PMC8799462; doi:10.1038/s41388-021-02115-7)
Supplement: Supplementary file 3 — Supplementary Table 2 [file 41388_2021_2115_MOESM3_ESM.docx]

**Table S2. Limiting Dilution Analysis of SCC25 in vitro**

| **Group** | **Cell Dose** | **Incidence** | **Frequency** | **P value (vs. NC)** |
| --- | --- | --- | --- | --- |
| NC | 100 | 24/24 | 1/23.91(1/38.47-1/14.94) |  |
|  | 10 | 8/24 |  |  |
| Cisplatin  Starvation  Hypoxia | 1  100  10  1  100  10  1  100  10  1 | 1/48  24/24  17/24  15/48  24/24  15/24  17/48  24/24  16/24  15/48 | 1/5.83(1/8.47-1/4.07)  1/6.25(1/9.13-1/4.34)  1/6.26(1/9.15-1/4.35) | 3.96e-06  1.08e-05  1.34e-05 |
